# Eco-evolutionary processes shaping floral nectar sugar composition

**DOI:** 10.1038/s41598-024-64755-5

**Published:** 2024-06-15

**Authors:** Yicong Liu, Susanne Dunker, Walter Durka, Christophe Dominik, Jonna M. Heuschele, Hanna Honchar, Petra Hoffmann, Martin Musche, Robert J. Paxton, Josef Settele, Oliver Schweiger

**Affiliations:** 1 Department of Community Ecology, Helmholtz-Centre for Environmental Research-UFZ, Halle (Saale), Germany; 2 Institute for Biology, Martin Luther University Halle-Wittenberg, Halle (Saale), Germany; 3 Department of Physiological Diversity, Helmholtz-Centre for Environmental Research-UFZ, Leipzig, Germany; 4 German Centre for Integrative Biodiversity Research (iDiv) Halle-Jena-Leipzig, Leipzig, Germany; 5 Department of Biodiversity and People, German Centre for Integrative Biodiversity Research-Jena-Leipzig, Leipzig, Germany; 6 Department of Conservation Biology and Social-Ecological Systems, Helmholtz Centre for Environmental Research-UFZ, Halle (Saale), Germany; 7 Department of Ecological Monitoring, Institute for Evolutionary Ecology, NAS Ukraine, Kyiv, Ukraine

**Keywords:** Evolution, Ecology, Evolutionary ecology, Macroecology

## Abstract

Floral nectar sugar composition is assumed to reflect the nutritional demands and foraging behaviour of pollinators, but the relative contributions of evolutionary and abiotic factors to nectar sugar composition remain largely unknown across the angiosperms. We compiled a comprehensive dataset on nectar sugar composition for 414 insect-pollinated plant species across central Europe, along with phylogeny, paleoclimate, flower morphology, and pollinator dietary demands, to disentangle their relative effects. We found that phylogeny was strongly related with nectar sucrose content, which increased with the phylogenetic age of plant families, but even more strongly with historic global surface temperature. Nectar sugar composition was also defined by floral morphology, though it was not related to our functional measure of pollinator dietary demands. However, specialist pollinators of current plant-pollinator networks predominantly visited plant species with sucrose-rich nectar. Our results suggest that both physiological mechanisms related to plant water balance and evolutionary effects related to paleoclimatic changes have shaped floral nectar sugar composition during the radiation and specialisation of plants and pollinators. As a consequence, the high velocity of current climate change may affect plant-pollinator interaction networks due to a conflicting combination of immediate physiological responses and phylogenetic conservatism.

## Introduction

Species interactions are an important component of the functioning of entire ecosystems^1^ and are subject to changes at evolutionary and ecological time scales^2^. Plant-pollinator interactions are of considerable relevance, given their ecological and economic importance^3^, particularly under the current global decline of pollinators^4^. Floral rewards are essential for structuring plant-pollinator interactions, in which nectar in particular plays a vital role in attracting pollinators. Nectar contains multiple nutrients, primarily in the form of carbohydrates—the disaccharide sucrose and the hexose monosaccharides glucose and fructose. Nectar sugar composition tends to be highly variable between plant species^5^ and can be subject to many drivers such as evolutionary processes shaped by pollinator consumer demands, flower morphology, and phylogenetic conservatism^6^. However, compared to other nectar traits (e.g. nectar volume and nectar sugar concentration) which vary with pollinator types^7^ and environmental variables such as light, water, soil conditions and temperature^8^, nectar sugar composition might be more invariant and species-specific^9^ (but see Herrera, et al.^10^).

Nectar sugar composition might be modulated by selective pressures through the energetic and nutritional requirements of pollinators. Pollinators with high energy demands, such as hummingbirds, lepidopterans and bees, prefer sucrose-rich nectars, whereas pollinators with low energy demands, such as bats and flies, tend to visit flowers with hexose-rich nectars^11^. Although sucrose-rich nectars are usually produced in lower volumes, they are more concentrated, whereas hexose-rich nectars are usually copious but diluted^12^ due to their higher osmolarity, which draws water from the plant tissue to the nectar^13^. The concentration and composition of nectar ingested by insects can also be critical for their water balance. For instance, large flying insects that consume diluted nectar can have difficulties to excrete excess water or have higher energy expenditure during foraging flights, indicating the need for matching sugar composition with species-specific water demands^14^.

Phylogeny might also contribute to variation in nectar sugar composition, potentially due to long-term changes in climatic conditions^15^. The appearance of nectaries in angiosperms dates back to the Late Cretaceous^16^ in response to prevalent global warming and consequent aridity^17^, which might have imposed constrains on the chemical-physical characteristics of plants, such as their water balance^18^. In addition, Sturm and Tang^19^ showed that the hydrolysis of sucrose to hexoses is more favourable under cold temperatures, resulting in higher hexose proportions at high latitudes or altitudes.

Nectar sugar composition is also linked to flower morphology^20^. For instance, deep and concealed flowers have sucrose-rich nectars associated with diminishing water loss through evaporation^21^. Shallow flowers with more exposed nectaries tend to have hexose-rich nectars which can better equilibrate with ambient humidity^22^ or better compensate evaporation by drawing water from surrounding plant tissue^23^. In addition, highly complex, bilaterally symmetrical flowers can be regarded as an adaptation to protect sucrose-rich nectars. This is usually reflected by interactions with specialist pollinators, while generalist pollinators tend to visit flowers with sucrose-poorer nectars of morphologically less complex flowers^11^.

Our understanding of the evolution of nectar sugar composition is still limited^24^. While most former studies have been restricted to single plant families, we rigorously measured and collected nectar sugar composition data by covering most of the major lineages of insect-pollinated European eudicot angiosperms. Here, we disentangle the relative importance of (i) phylogenetic effects in relation to paleoclimate (ii) flower morphology, and (iii) selective pressures exerted by pollinator dietary demands on nectar sugar composition. We expected that all three aspects are not mutually exclusive but rather that they act in combination. Better knowledge about their relative importance is needed to understand and, moreover, predict the processes structuring plant-pollinator networks, particularly under changing environmental conditions.

## Results

### Nectar sugar composition

**Figure 1:**
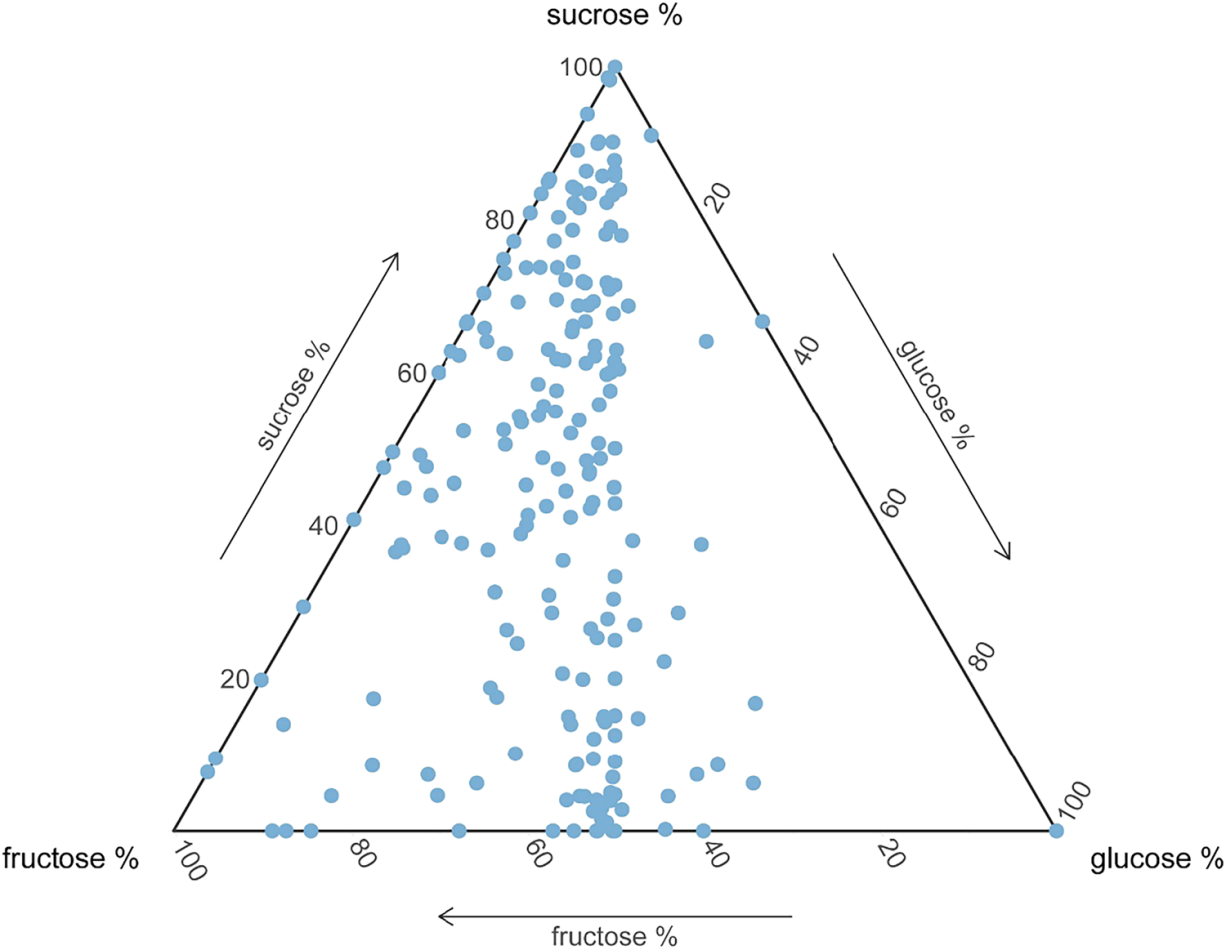
Ternary diagram of nectar sugar composition of 414 Central European plant species.

Our measure of the sucrose content of nectar with respect to total sugar content, which we term *the proportion of sucrose in nectar*, varied substantially, ranging from 0 to 100%, but floral nectar of most plant species (71%) was dominated by hexoses. The separation of hexose sugar composition along the fructose axis indicated that the proportion of fructose was often greater (48% of the species) or equal (48% of the species) to that of glucose (Fig. 1).

### Proportion of sucrose in nectar

**Figure 2:**
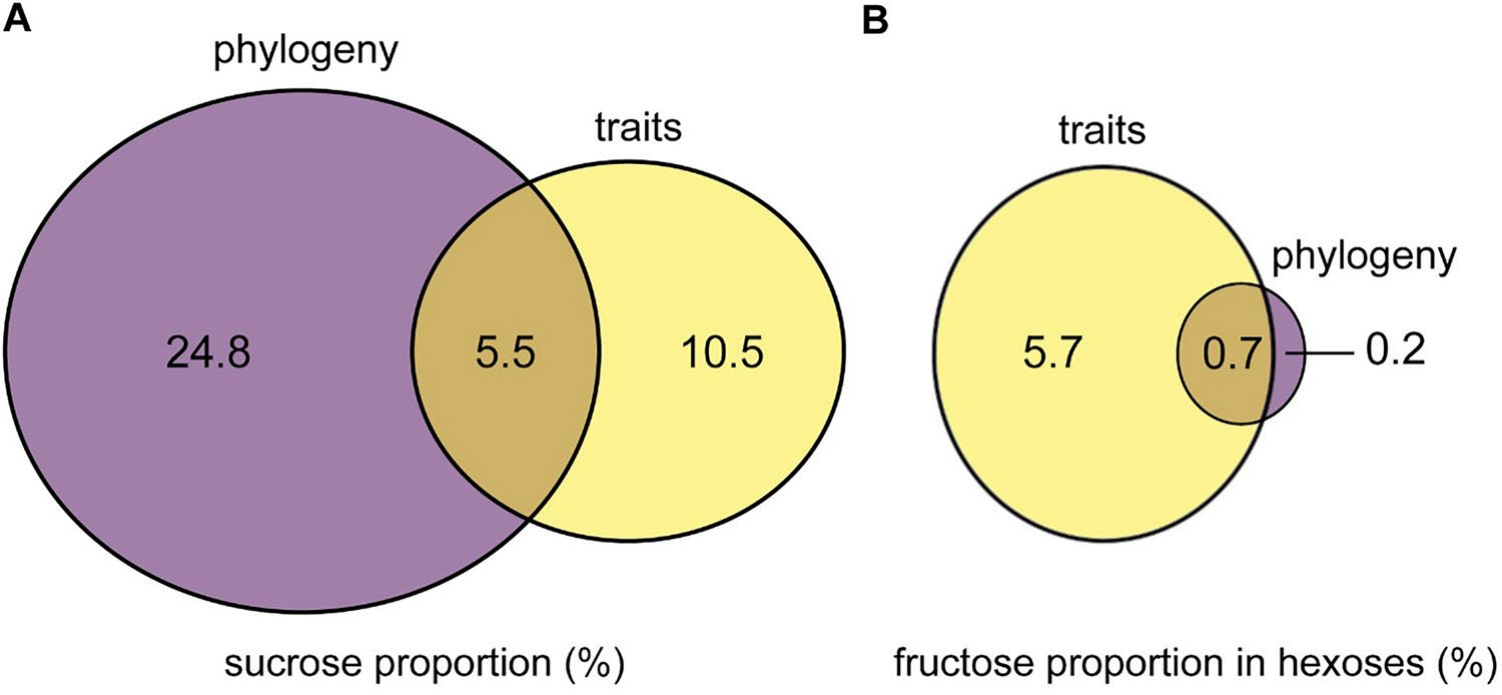
Venn diagrams representing partition of the variance in the proportion of sucrose in nectar (**A**) and variance in the proportion of fructose within hexoses (**B**) explained by trait (yellow circle), phylogeny (purple circle) and phylogenetically structured traits (overlapped circles). Note: the sizes of the ovals are not strictly proportional.

Sixteen phylogenetic eigenvectors were selected in an initial phylogenetic eigenvector regression (PVR) analysis to explore a plant phylogenetic signal explaining the proportion of sucrose in nectar. After further simplification of a full model (including phylogenetic and trait variables), 10 phylogenetic eigenvectors, corolla tube length, and flower symmetry remained in the set of best models. Pollination type, flower type, and flower colour were not correlated with the proportion of sucrose in nectar. Taken together in one model, the explanatory power of the 10 phylogenetic eigenvectors, corolla tube length, and flower symmetry was high (40.8%) for variation in the proportion of sucrose in nectar. The largest part of the variation was explained by phylogeny (24.8%), while floral traits explained 10.5% of the variation, and the common fraction of variation shared by phylogeny and floral traits was 5.5% (Fig. 2A).

**Figure 3:**
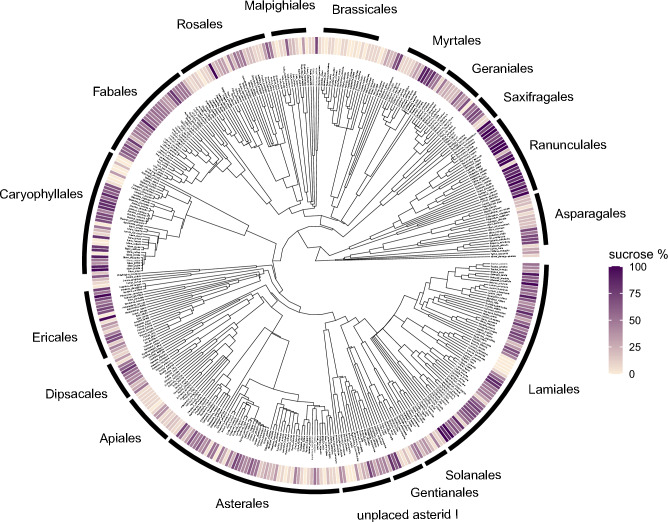
Trait mapping of the proportion of sucrose in nectar on the phylogenetic tree of 414 plant species.

**Figure 4:**
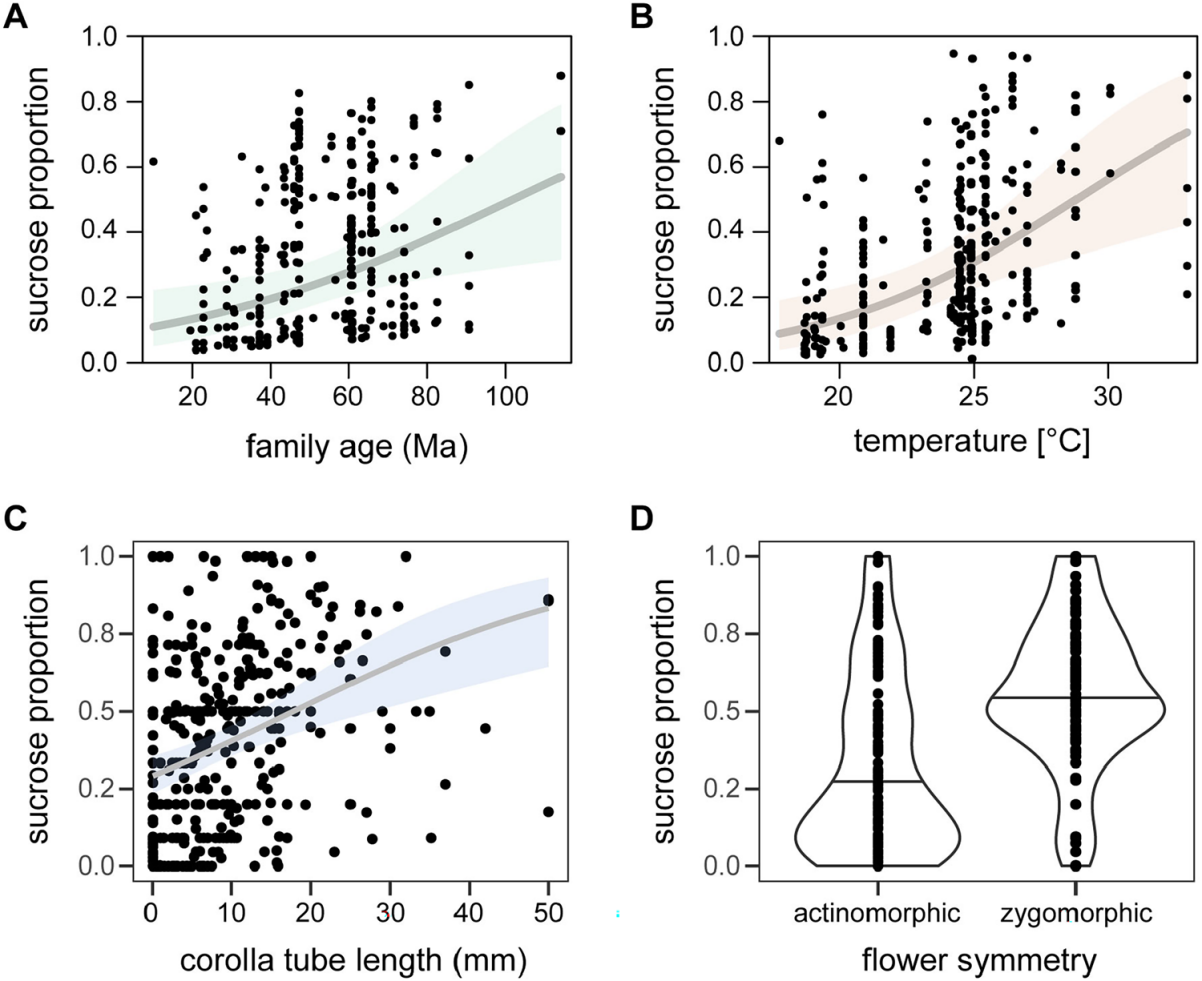
Relationship between the proportion of sucrose in nectar and plant family age modelled with plant phylogenetic order as random effect (**A**), and global mean annual surface temperature during the time of the origin of plant families, i.e. from Cretaceous until nowadays (**B**), plant corolla tube length (**C**), and flower symmetry (**D**). Solid line indicates the predicted relationship (at the response scale) and shaded area represents the 95% confidence intervals.

The proportion of sucrose in nectar was phylogenetically conserved (Fig. 3) and showed a positive relationship with the phylogenetic age of the plant family (slope = 0.014, p < 0.001, AIC = 456.56), but not with the age of the plant order (p = 0.117). Including plant family nested in plant order as random effects increased model performance and the strength of the positive relationship (Fig. 4A, slope = 0.027, p = 0.008, AIC = 432.61). Related to this, the proportion of sucrose in nectar also increased with the global mean annual surface temperature during the time of origin of the respective plant families with even higher explanatory power (Fig. 4B, p < 0.001, AIC = 429.34). The proportion of sucrose in nectar also increased with flower corolla tube length (Fig. 4C) and was higher in zygomorphic flowers than in actinomorphic flowers (Fig. 4D).

### Proportion of fructose within hexoses

In contrast to the proportion of sucrose in nectar, the explained variance in the proportion of fructose within hexoses was low (6.6%), but the explanatory power of traits (5.7%) was considerably higher than that of phylogenetic relationships (0.2%; Fig. 2B). The common fraction of variation shared by phylogeny and traits was 0.7%, indicating that the largest impact of phylogeny was via phylogenetically structured floral traits. We did not find a relationship of the proportion of fructose within hexoses to clade age. Similar to the proportion of sucrose, the proportion of fructose within hexoses increased with flower corolla tube length (Fig. S3A) and was higher in zygomorphic flowers compared to actinomorphic flowers (Fig. S3B).

### Species-level specialisation

**Figure 5:**
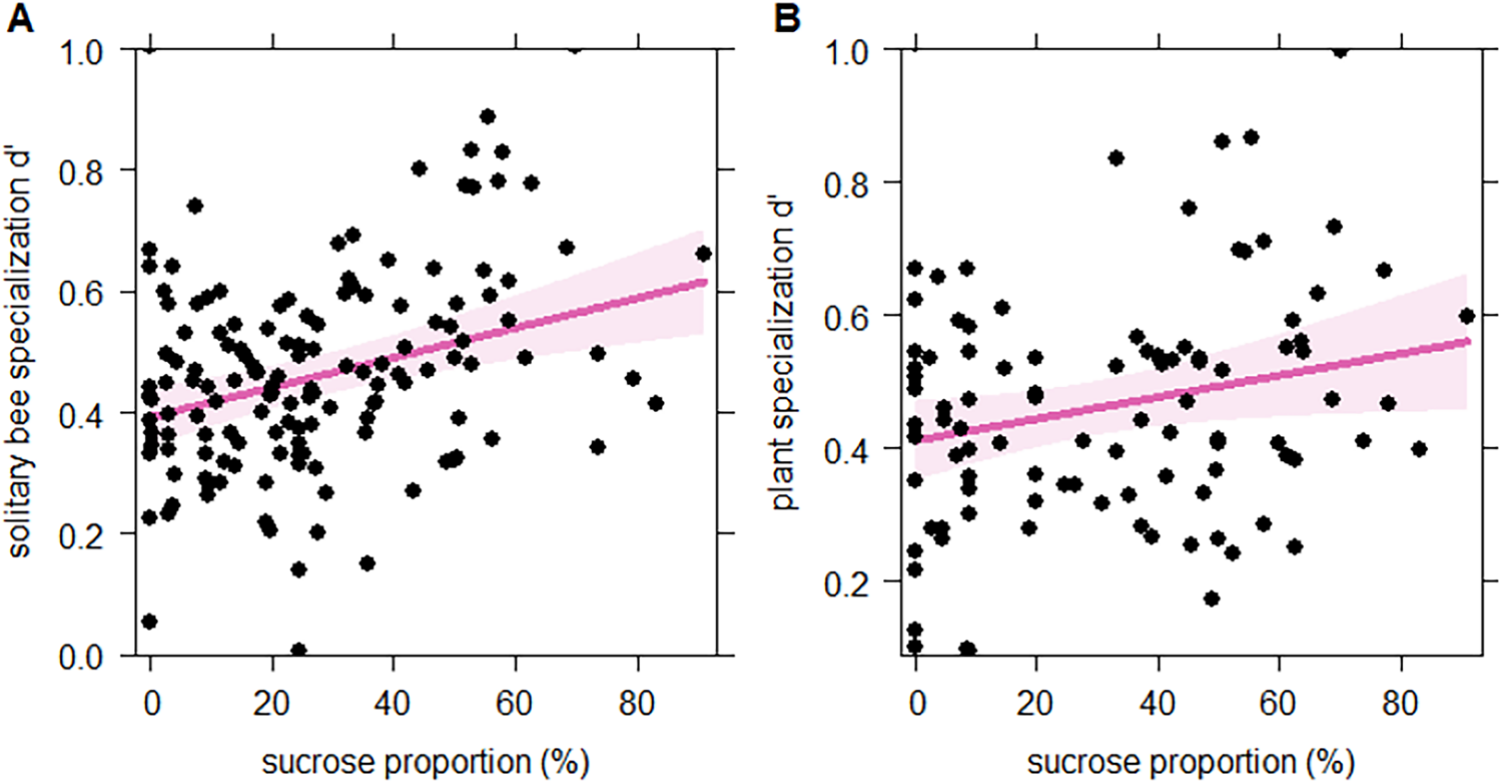
The effect of the proportion of sucrose in nectar on the species-level specialisation (d`) of solitary bees (**A**) and respective plant species (**B**). The solid line indicates the predicted relationship and the shaded area represents the 95% confidence intervals.

We recorded 4676 plant-pollinator interactions in total, including 2013 plant-solitary bee interactions, 1544 plant-bumble bee interactions, 502 plant-hoverfly interactions and 617 plant- honey bee interactions. For the entire meta-network, we did not find a relationship between the proportion of sucrose in nectar and plant specialisation (d΄) after model simplification, but a significant but weak relationship between the proportion of sucrose in nectar and pollinator specialisation (d΄; p < 0.01, marginal R^2^ = 0.05, conditional R^2^ = 0.08). For plant-solitary bee networks, we found a positive relationship between the proportion of sucrose in nectar and the specialisation of solitary bees (Fig. 5A, p < 0.001, marginal R^2^ = 0.092, conditional R^2^ = 0.11) and of plant species (Fig. 5B, p = 0.035, marginal R^2^ = 0.049, conditional R^2^ = 0.69), i.e. high specialisation was related to sucrose-rich nectars for both solitary bees and plants. We did not find any relationship between the proportion of sucrose in nectar and the specialisation of bumble bees and hover flies.

## Discussion

Our results elucidate the effects of phylogeny, plant morphological traits, and dietary specialisation of pollinators on nectar sugar composition across Central European eudicot angiosperms. Phylogenetic relatedness played the most critical role in the proportion of sucrose in nectar, which decreased considerably from the early Late Cretaceous (94 Ma–86 Ma) until now, as inferred from the phylogenetic age of the plant family. However, this relationship was even stronger with decreasing global mean annual surface temperatures. Flower morphology such as corolla tube length and symmetry had relatively high power to explain the proportion of sucrose in nectar. We did not find a strong relationship between nectar sugar composition and a plant’s main pollinator group but, at a species-level resolution, the specialisation of pollinators and plants increased with the proportion of sucrose in nectar.

The proportion of sucrose in nectar constantly decreased throughout the evolution of plant families, displaying high proportions in ancient families such as the Papaveraceae (85%, ca. 114 Ma) and the Ranunculaceae (74%, ca. 65 Ma) and particularly low sucrose proportions in families more recently evolved, such as the Asteraceae (27%, ca. 37 Ma) or the Violaceae (17%, ca. 21 Ma). This decline coincides with a more or less constant decrease in global mean annual surface temperature. An important role of temperature was suggested by a better model fit compared to a simple temporal dependency.

Two non-mutually exclusive mechanisms might explain the impact of temperature, and paleoclimate in general, on the evolution of nectar sugar composition: (i) direct impacts in relation to plant water balance; and (ii) indirect effects on plant and pollinator radiation and specialisation. In spite of the general warm and humid greenhouse conditions during the Cretaceous (mean annual temperature up to 33 °C), a steady shift towards significantly drier climates^25^ gave rise to the radiation of angiosperms, particularly in mid-latitude ecosystems^26^. Such drier conditions can challenge a plant’s water balance and generate selection pressures against hexose-rich nectars^18^ since they are more water-demanding than sucrose-rich nectars^24,27^. As a consequence, sucrose-rich nectars are likely to provide an evolutionary advantage under warmer and drier conditions, which is supported by the current dominance of sucrose-rich nectars under arid conditions^28^.

Although evidence was rather weak, our results for solitary wild bees indicate potential selective pressures exerted by dietary demands of pollinators because specialised pollinators were associated with sucrose-rich nectars while generalist pollinators were more associated with sucrose-poor nectars, the latter supporting previous results of Abrahamczyk, et al.^24^. The temporal patterns, in particular the dependency of nectar sugar composition on temperature, and the link between sucrose-rich nectars and specialisation of both solitary wild bees and plants, suggest a relatively higher level of specialisation during the particularly warm phases of major co-radiation of both flowering plants and flower-visiting pollinators in the early Late Cretaceous^29,30^, followed by a decrease in specialisation with time and temperature (present day: 13 °C mean annual temperature). Phylogenetic conservatism of these evolutionary processes can be inferred by similar present-day large-scale patterns of plant-pollinator networks, with higher levels of specialisation under warmer conditions across latitudinal and elevational gradients^31^, and stronger impacts of historical climate change compared to contemporary climate^32^.

Taken together, our results provide support for the combined effects of both climate-driven selection pressures, reflecting adaptation of a plant to its water balance, and adaptation to the level of specialisation, as seen in the proportion of sucrose in its nectar. Before the mid-Cretaceous, the major pollinators comprised generalists such as Lepidoptera, Coleoptera and Diptera^33^. Increasing water balance constraints under increasing temperatures and drier climatic conditions during the early Late Cretaceous likely caused a shift from hexose-rich nectars to sucrose-rich nectars^18^. The evolution of novel and energy-rich high sucrose nectar, accelerated evolutionary rates facilitated by high temperatures^34^, increasing species richness, and the potential of switching to novel hosts^35^, likely facilitated the speciation of many pollinators^31^ and plants as well as their reciprocal specialisation^36^. In addition to our results, initially high levels of pollinator specialisation are also supported by analyses of fossil pollen^37^ and narrow host-plant specialisation in many basal pollinator lineages^38^.

Decreasing temperatures and less arid conditions^39^ likely relaxed plant water balance constraints over time and thus might have paved the way for the evolution of more generalised plant-pollinator interactions supported by increased proportion of hexose in nectar, which can be digested more efficiently by (some) generalist pollinators^22^. A major cause favouring the evolution of generalised plant-pollinator interactions might be declines in species richness and abundance with cooler temperatures, as indicated by current large-scale patterns^40^, which relax interspecific competition^41^ and also might decrease the predictability of interactions with specialised partners. For plants, generalisation can decrease the dependence on specific pollinators and thus stabilize pollination^42^, particularly when the availability of the most effective pollinator is hard to predict^43^. For pollinators, generalisation can be related to the costs of foraging and can be of particular advantage under energy-restricted conditions, e.g. when floral resources are less abundant^44^ or under cooler temperatures^45^. This might also have consequences under current and future climate change, where the particularly high velocity of change might drastically limit the options for local adaptation. As a consequence, nectar sugar composition might no longer match the requirements of pollinators well^46^, potentially affecting plant-pollinator network structures and in turn plant pollination and reproduction. However, phenotypic plasticity, e.g. via (selective) reabsorption of sugars or changes in pollinator foraging behaviour^22^, might buffer such negative effects, but the efficiency of such a buffering effect remains to be investigated.

In contrast to our expectation, we did not find evidence of adaptive processes of nectar evolution according to plants’ predominant pollinator groups, although we covered most of the major insect pollinated plant lineages of Europe. This concurs with studies showing that floral traits or pollination syndromes are poor predictors of flower visitors^47^. Our study shows that a coarse functional classification of flower types might obscure indications of selective pressures on nectar sugar composition via pollinator dietary demands.

However, applying an ecological concept of specialisation revealed a small but significant relationship between species-specific specialisation of pollinators (at least for solitary bees), plants and their nectar sugar composition. The results for solitary bees confirm the findings of Abrahamczyk, et al.^24^, but also highlight the need for refined, ecological measures of specialisation at higher taxonomic resolution when analysing a restricted set of plant-pollinator interactions, such as for the exclusively insect-pollinated plants in Europe.

Although significant, the effect of the proportion of sucrose in nectar on specialisation of solitary bees and plants was comparably small, which might be indicative of high plasticity in the foraging behaviour of pollinators^22^. Another reason might be potential intraspecific spatio-temporal variability in nectar sugar composition^8^, which was covered only to a limited extent by our study design and might have introduced random noise. This suggests that the actual relationship might be even stronger. Such potential noise might also have obscured a relationship for bumble bees and hover flies, for which we already had limited power to detect specialisation (13 bumble bee, 24 hover fly species).

Flower morphology showed the expected relationship with nectar sugar composition. Zygomorphic and long-tubed flowers with concealed nectar had a higher proportion of sucrose in nectar than actinomorphic and short-tubed flowers with exposed nectar. These results corroborate the findings of previous studies^48,49^. Although the effects of floral morphological traits were largely independent of phylogenetic relationships, one third of the variation in the proportion of sucrose in nectar explained by flower morphology was due to phylogenetically structured traits. This indicates a strong evolutionary pressure linking flower morphology and nectar sugar composition, likely within the context of protection against evaporation relevant for a plant’s water balance^50^ and to provide nectar in an adequate form for pollinators, i.e. not too diluted or too viscous^51^.

Since the hexoses glucose and fructose are generated via hydrolysis of the disaccharide sucrose by cell-wall invertases during nectar secretion, a 1:1 ratio of glucose and fructose might be expected. This was the case for approximately half (190/397) of the plant species, but almost all of the remaining 52% had higher proportions of fructose than glucose. Uneven proportions of both hexose sugars might be caused by less-well understood reabsorption mechanisms contributing to homeostasis or recovery of the investment in nectar^22^. Although the effect was small, the proportion of fructose (relative to glucose) depended in a similar way on flower morphology as the proportion of sucrose, with fructose-rich nectar related to zygomorphic and long tubed flowers. This might again be related to plant water balance and reduced evaporation via increased surface tension. The surface tension of sugar solutions decreases, and evaporation consequently increases, in the following order: glucose, fructose, sucrose^52,53^. This might explain the similar, but less pronounced, relationship between the proportion of fructose within hexoses and flower morphology, as we found for plants with sucrose-rich nectars. However, the relationships for the proportion of fructose within hexoses were weak and did not show a marked phylogenetic signal, indicating either lower ecological and evolutionary relevance, or microbial activities that can also contribute to the asymmetric nature of a plant’s nectar sugar composition^54^. One methodological issue might be that we sampled unbagged flowers which therefore may have been visited by pollinators, which in turn may have infected nectar with microbes, thereby changing the nectar sugar profile^55^. However, we do not consider this a major problem because there was a good match between our own and literature data; mean values of nectar composition were consistent across the different sources which were used for the analyses (Fig. S1 and Fig.S2). Furthermore, excluding our data from the analyses led to very similar results for the overall pattern of nectar sugar composition (Fig. S4S5 and S6), supporting the idea that our empirical data are valid.

Nectar sugar composition does not cover the entire spectrum of floral rewards for pollinators^56^. To assess evolutionary mechanisms of plant-pollinator interactions more fully and to improve our predictive abilities, future studies might expand the focus on the total amount of nectar, other chemical components of nectar such as mineral nutrients, amino acids or volatile compounds, the amount and quality of pollen, potential trade-offs relative to their metabolic costs, and evolutionary aspects of phyto-chemical mechanisms, e.g. related to the recently discovered sugar transporter SWEET9 and apoplasmic invertase^57^ or photosynthesis-related temporal availability of nectar^58^.

## Conclusions

Our study disentangled the contributions of evolutionary and ecological processes on nectar sugar composition, which is essential for understanding the potential mechanisms underlying current plant-pollinator interactions. Our results indicate strong phylogenetic conservatism in nectar sugar composition, likely driven by selective pressures related to plant water balance. While evidence for selective pressures by pollinator dietary demands was small, potential evolutionary legacy effects are indicated to be still visible in current plant-pollinator networks, where in particular plants with a high proportion of sucrose in nectar are more readily visited by specialised pollinators. Strong phylogenetic conservatism together with a higher level of specialisation of both plants with a higher proportion of sucrose in nectar and their pollinators might put additional pressure on current plant-pollinator networks, especially those in drier and warmer regions, particularly under ongoing biodiversity loss and pollinator decline.

## Methods

We collected a comprehensive data set on nectar sugar composition for 414 angiosperms across Central Europe together with information on plant phylogeny, floral traits, and a functional classification of pollinator dietary demands. We used a hierarchical variance partitioning approach to disentangle and quantify the relative importance of each of these traits. We further assessed changes in nectar sugar composition during plant evolution and related them to respective global temperature trajectories. To improve our analyses of the relationship between nectar sugar composition and pollinator dietary demands, we extracted species-level measures of specialisation for both plants and pollinators from current plant-pollinator networks, refining the approach based on a functional classification scheme.

Our 414 European plant species belonged to 28 orders and 67 families. All 414 species were used for analyses of the sucrose content of nectar as a proportion of the total sugars in nectar, which we term *the proportion of sucrose in nectar*, and 397 species were used for the analysis of the fructose content of nectar as a proportion of all hexoses in nectar, which we define as *the proportion of fructose within hexoses* (17 species had 100% sucrose). For all 414 species, data on flower type were obtained from the literature. Data on nectar sugar composition were based on own measurements for 89 plant species, and complemented by literature records for the remaining 325 species. Data on floral morphology were obtained empirically for 388 species, complemented with data from the literature for 26 species. We also used empirical data on local plant-pollinator networks, covering 123 plant species (all within the set of 414 species) and 188 pollinator species (150 solitary bees, 13 bumble bees, 24 hoverfly species, *Apis mellifera*).

### Nectar collection and sugar composition

We collected the nectar standing crop from unbagged individual flowers of 89 flowering plant species between May and August 2021 at 16 sites (also used for plant-pollinator network assessments; see below) in the federal state of Saxony-Anhalt, Germany, covering a rural to urban gradient (Table S2). We used 0.5, 1, or 5 µl glass micro-capillary tubes (Hirschmann^®^ minicaps^®^), depending on the flower size and nectar produced. For 32 plant species we directly extracted the nectar with the micro-capillary while, for the remaining 57 species, nectar amount was too low for direct extraction. We therefore rinsed individual flowers with 1–3 μl of distilled water, which we added to the nectaries and then collected it 1 min after application as a diluted solution. We cumulatively sampled as many open flowers per plant species (randomly across a site) to reach the minimum volume required for subsequent chemical analyses (0.08 μl). To address intraspecific variation, we sampled on average 3.1 flowers per plant species (ranging from 1 to 10) from up to three sites and up to three sampling dates.

Nectar samples were prepared and carbohydrate was measured based on methods of Witt, et al.^59^, performed with a high-performance anion exchange chromatography system (see Supporting Information for details). Multiple nectar samples per plant species (average of 3.1, range from 1 to 10) were analysed separately and sugar composition was then averaged for subsequent statistical analysis (Table S3 will be deposited in a public repository).

### Nectar data from the literature

To complement our nectar database, we compiled nectar sugar composition data from 7 publications^5,9,24,49,60–62^ resulting in data from 325 additional plant species. Since nectar sugar composition was qualitatively described in Percival^5^, we redefined these subjective assessments as semi-quantitative values (see Supporting Information).

### Phylogeny and historic temperatures

The phylogeny of the Central European flora was extracted from the dated phylogeny “DaPhnE”^63^. Information on plant phylogenetic age, defined as the age of the most recent common ancestor of all living species of the clade, was extracted at both the family and order levels. To relate the evolution of nectar sugar composition to long-term temperature changes, we extracted historic global mean annual surface temperatures from Tierney, et al.^64^, covering the time from 100 Ma until now in steps of 0.2 My. Respective temperatures during the origin of the plant families were calculated by linear interpolation.

### Plant trait data

At the 16 sites used for nectar sampling, we measured flower corolla tube length as the distance between corolla insertion where the nectary is accessible and the beginning of corolla lobes where a flower visitor can land, using an average of 5 flowers for 388 species (Table S3). For the remaining 26 plant species, we extracted the relevant data from Cappellari, et al.^65^.

Plant species were assigned to two categories based on the number of floral symmetry axes, i.e. actinomorphic (radially symmetric) and zygomorphic (bilaterally symmetric). We assigned floral symmetry based on the entire inflorescence for Asteraceae only. From BiolFlor^66^, we extracted nine levels of flower colour (white, yellow, violet, purple, pink, red, blue, green and brown) and ten categories of flower type (disk-bowl, funnel, bell, stalk-disk, lip, flag, head, brush and trap flowers; see Supporting Information for details).

To assess potential evolutionary processes between nectar sugar composition and groups of pollinators, we used a functional classification system of flower pollination types sensu ^67^. The classification was based on Müller’s^68^ initial flower types, considering the most predominant pollinator groups visiting the flowering plants, but updated to consider contemporary refinements, as follows. Waser, et al.^69^ pointed to generalisation rather than specialisation in current networks. Consequently, we combined 11 initial categories into the new group “generalised flowers”. However, Fenster, et al.^70^ stated that, despite the widespread occurrence of generalisation, pollination syndromes can nevertheless help us to understand floral variation and thus are meaningful classifications when treated with care. We therefore combined 23 classes of Müller^68^ into 7 broader pollination types: “hymenopteran flowers”, “bee flowers”, “bumble bee flowers”, “butterfly flowers”, “moth flowers”, “fly flowers”, and ”others” (see Supporting Information).

### Plant-pollinator network sampling

In addition to the functional classification of flower types, we identified the level of ecological specialisation sensu^67^ for both flowering plant and pollinator species based on empirical plant-pollinator networks. Ideally, pollination effectiveness of each flower visitor should combine visitation frequency and pollen transfer^71^ but, lacking the latter, we relied on visitation networks as a reasonable proxy of pollination^72^. Networks were sampled three times in May, June-July and August in 2021 at the same 16 sites from which samples for nectar analysis and flower morphology were taken (Table S2), resulting in 48 networks. At each site, flower-visiting bees (Anthophila) and hover flies (Syrphidae) which touched the reproductive parts of the visited flowers with any part of their body were sampled by netting along a 1 km transect for a total of 120 min (excluding handling time) from morning to afternoon (09.00–18.00) under dry, warm (> 14 ˚C) and low-wind weather conditions. The combination of flower visitor and respective flower were recorded as an “interaction”. Visited plant species were identified in situ using Flora Incognita^73^. Insects were conserved and identified to species level by experts, complemented with DNA barcoding in the few cases of morphological uncertainty. Species were sampled in accordance with the nature protection laws of Sachsen-Anhalt with permission of Sachsen-Anhalt’s environmental bureau: licence number RL-0580.

### Statistical analysis

All analyses were conducted in R v. 4.2.3^74^. We calculated the proportion of sucrose (our *proportion of sucrose in nectar*) as the molarity of sucrose versus the molarity of all three sugars (sucrose, glucose and fructose), and the *proportion of fructose within hexoses* (glucose and fructose). To assess a potential bias by combining different data sources for nectar sugar composition and by using unbagged flowers for our dataset, we first compared the the proportion of sucrose in nectar for the same species coming from different literature sources and our dataset using a two-sample Wilcoxon rank sum tests. Ignoring datasets with only up to three species, we found that there was no significant difference between our empirical and published data (Fig. S1). To assess the general comparability of the different datasets relative to the sugar composition values we were actually using, namely: the mean values across plant species and with information on nectar sugar composition from multiple sources, we related the original values per source to the mean values across different sources. For this, values coming from a single source were ignored. A small scatter around the one-to-one line (R^2^ = 0.81; Fig S2) indicated high comparability across the different datasets and no systematic bias.

To partition the variation in nectar sugar composition between plant phylogeny, functional traits (i.e. flower corolla tube length, flower symmetry, flower colour, pollination type and flower type) and phylogenetically structured traits, we used phylogenetic eigenvector regression (PVR) analyses^75,76^.

First, we generated eigenvectors of the pairwise phylogenetic distance matrix after a double-centred transformation based on principal coordinates analysis (PCoA)^76^. Second, we regressed the eigenvectors against sucrose proportion and proportion of fructose relative to glucose, and identified relevant phylogenetic eigenvectors with a stepwise selection procedure based on the Akaike information criterion (AIC). Since sugar composition was measured as proportions, we modified the PVR function in the R package PVR^77^ to account for a binomial error distribution. For the proportion of fructose relative to glucose, no eigenvector was retained after AIC-based selection. We therefore included all floral traits in addition to all eigenvectors generated from the first step to strengthen the explanatory power of the model. As a result, only the first eigenvector was retained in our model. For comparison, we selected eigenvectors based on Moran’s I^75^. Both approaches supported the need to retain the first eigenvector in PVR analysis.

Selected phylogenetic eigenvectors were then used as predictor variables together with floral traits in generalised linear models (GLMs) with a binomial error distribution and a logit-link function to explain the proportions of sugars in nectar. We simplified the initial models by calculating all possible variable combinations and selecting the best set of models with a delta AIC < 2 using the R package MuMIn^78^. For subsequent variance partitioning, we considered all explanatory variables occurring in the set of best models.

Finally, the explanation of variation in sugar proportions was partitioned between three components: the trait matrix, the phylogenetic matrix, and their overlap (phylogenetically structured traits^79^) using likelihood-ratio based adjusted pseudo-R^2^^80^.

To assess the evolutionary depth of a potential phylogenetic signal in floral nectar sugar, we used GLMs with a binomial error distribution and a logit link function in the R package lme4^81^. We related sucrose proportion to the respective ages of the plant order and family in two separate models. To correct for the nested phylogenetic relatedness between family and order, we also performed generalised linear mixed effects models (GLMMs), treating the age of the plant family as a fixed factor and the plant order as a random effect.

To further deepen our understanding of the phylogenetic signal, we interpolated the paleotemperature for the time of the origin of the respective plant family on the basis of the global mean surface temperature from the past 100 Mya^64^. We developed GLMMs with a binomial error distribution and a logit link, with nectar sugar proportion as response variable, paleotemperature and plant traits as predictors and corrected for phylogenetic relationships by specifying random effects of plant family nested within plant order.

To identify the effects of nectar sucrose proportion and plant morphological traits on species-specific specialisation of plants and pollinators, in contrast to a functional classification based on flower types only, we generated a meta-network to capture the properties of plant–pollinator interactions emerging at broader spatial and temporal scales^82^. In such a meta-network, impacts of gene flow, geographical variation in selection, and coevolutionary effects are reflected through their potential consequences for trait evolution^83^. To do so, we pooled all empirical network data across the 16 sites and three sampling rounds and focused on the aggregated interaction network. To account for differences in overall abundances and total number of interactions among the different networks, we standardised pairwise interaction frequencies by dividing them by the total number of observed interactions per network before pooling them, i.e. taking the average of the interactions between pollinators and flowering plants. These proportional data were then rescaled to the actual range of interactions by multiplying them by the total number of interactions within the entire meta-network, and including a small correction factor. From this meta-network, we calculated species-level specialisation (d΄) of plants and pollinators using the R package bipartite^84^. Species-level specialisation (d΄) measures how specialised a given pollinator or plant species is in terms of both proportional resource utilisation and availability; it ranged from 0 for most generalised to 1 for most specialised^85^. We then used linear mixed-effects models to relate specialisation of plant species to nectar sucrose proportion, and specialisation of pollinator species to the average sucrose proportion of the plants they visited, weighted by the number of visits. We also included corolla tube length weighted by the number of interactions, and symmetry represented by the proportion of zygomorphic flowers for pollinator species as predictor variables, and taxonomic group of pollinators and plants, i.e. genus nested in family, as a random effect. For model simplification, we selected the best model based on a multimodel inference approach using the R package MuMIn^78^. We used the entire network to assess the specialisation of plants (122 species) and repeated this analysis for networks defined by all pollinators including *Apis mellifera* (188 species), and separately for networks defined by bumble bees (13 species), solitary wild bees (150 species), and hoverflies (24 species). For specialisation of bumble bees, we used generalised linear regression models without any random effect, and for hoverflies we included genus as a random effect.

### Supplementary Information

Supplementary Information.

Supplementary Table S3.

## Data Availability

All data generated or analysed during this study are included in this published article [and its supplementary information files].
