# Supplementary material for: Eco-evolutionary processes shaping floral nectar sugar composition
Source: Sci Rep. 2024 Jun 15;14:13856. doi: 10.1038/s41598-024-64755-5 (PMC11180116; doi:10.1038/s41598-024-64755-5)
Supplement: Supplementary file 1 — Supplementary Information. [file 41598_2024_64755_MOESM1_ESM.docx]

**Eco-evolutionary processes shaping floral nectar sugar composition**

**Supporting information**

**Supplementary methods**

**Nectar sugar composition**

One hundred µl of pure water was added to freeze-dried nectar pellets, carefully resuspended, and then vortexed. An additional dilution step took place by extracting 20 or 40 µl and further resuspension with 1980 µl or 1960 µl of pure water, respectively. Lower dilution was applied to samples with the lowest concentrations. All diluted samples were filtered with sterile 0.2 µm PVDF syringe pre-filters (MACHEREY-NAGEL, Germany). For carbohydrate analysis, extracted samples were subjected to a high-performance anion exchange chromatography system ICS-5000 with a pulsed amperometric detector (Thermo Fisher Scientific, Dreieich, Germany). In the mobile phase, eluents (12 mM and 150 mM NaOH) were manually prepared from 50 % (w/w) NaOH (FisherChemical). A gradient program was used at a flow rate of 0.2 mL min-1 (Table S1). A Dionex CarboPac PA210-4 µm, 2 x 150 mm column and a Dionex CarboPac Guard PA210-4 µm, 2 x 30 mm were used as stationary phases (Thermo Fisher Scientific, Dreieich, Germany). The sample compartment was cooled to 20 °C and the column compartment was heated to 30 °C. All samples were injected as a volume of 2.5 µl. Detection was performed using an electrochemical detector with a disposable Au-electrode and a quadrupole pulse waveform at a sampling rate of 2 Hz. For carbohydrate quantification, calibration curves were measured for D-(-)-arabinose (used as an internal standard), D-(+)-glucose, sucrose, and D-(-)-fructose (SigmaAldrich) within a range of 1-50 mg l-1 using the software Chromeleon (V7.2.6).

**Nectar data from literature**

In Percival (1961), nectar sugar composition was qualitatively described and designated by letters. Specifically, capital letters meant plenty of sugar, lower-case letters indicated that there was only a trace of sugar. Bold type meant a strongly dominant sugar, italic type indicated a slight preponderance. We redefined these subjective assessments as semi-quantitative values 2, 1.5, 1, 0.3 and 0.2 for those five levels, i.e. capital bold, capital italic, capital, lower-case italic and lower-case letters, on the basis of quantitative data from publications as well as empirical data of the same plant species. After transformation, we calculated the mean proportion of sugars in nectar per plant species. The proportions of sucrose in nectar from different data sources were compared visually (Fig. S1 and Fig. S2).


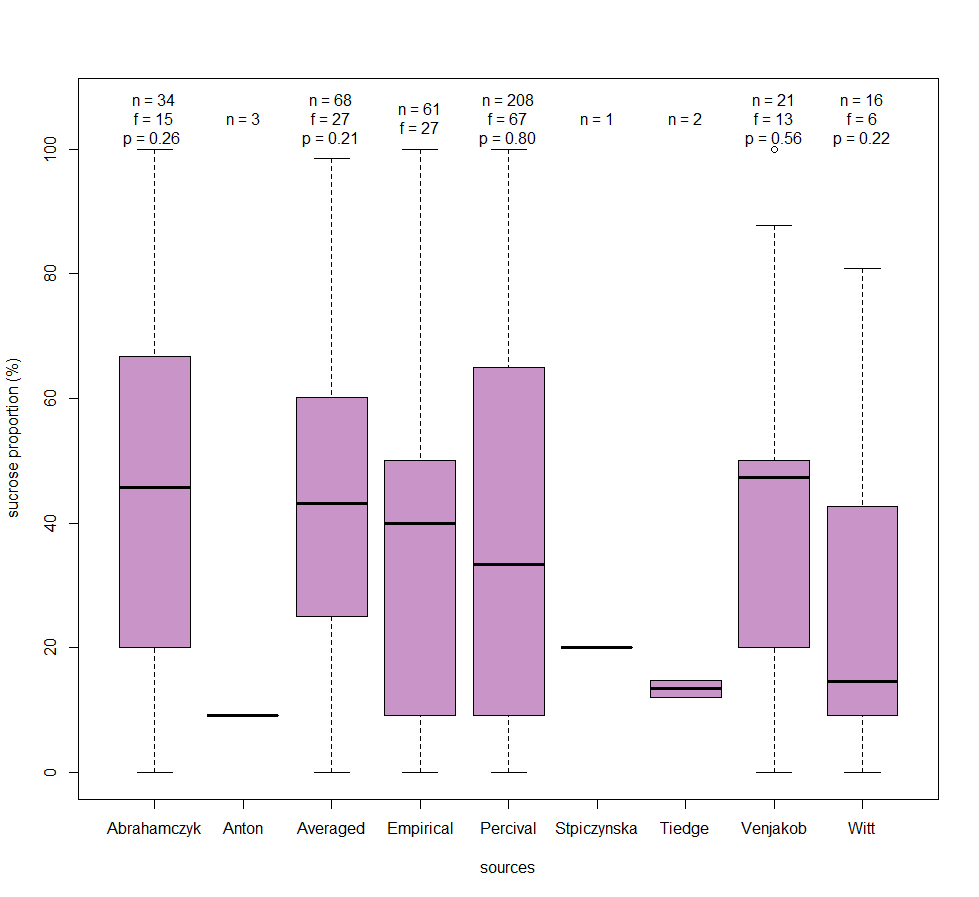


**Fig. S1** Boxplot of the proportion of sucrose in nectar from different sources for all species used in our analysis. ‘Averaged’ indicates the set of species for which we averaged the proportion of sucrose in nectar across two or more data sources. ‘n’ and ’f’ indicate the number of species and families used in the analysis, respectively. P values are based on two-sample Wilcoxon rank sum tests.


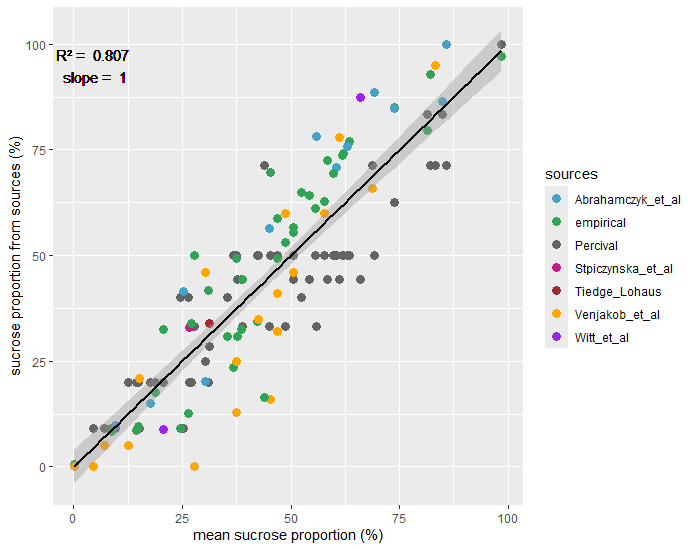


**Fig. S2** Relationship between the proportion of sucrose in nectar from different sources and mean values for each species that were used in the analysis after removing plant species whose mean values were directly used from a single source. Solid line indicates the relationship and shaded area represents the 95% confidence intervals.

**Flower type**

We recorded and classified flower types after Kugler (1970) into ten categories according to BiolFlor (Klotz *et al.* 2002): (1) disk-bowl (slightly concave); (2) funnel (an upward-facing funnel-shaped flower that the insect enters with much of, or its entire, body); (3) bell (a downward-facing bell-shaped flower that the insect enters with much of, or its entire, body, and not only its proboscis); (4) stalk-disk (a flower with a flattened part abruptly arising on a tubular stalk); (5) lip (a flower with an extended lip used by visiting insects as a landing platform); (6) flag (the “butterfly”-shaped flower of the Fabaceae and Polygalaceae); (8) head (a densely-packed flower aggregation with a more or less spherical or flat appearance; (9) brush (a single flower or a flower aggregation with numerous protruding anthers); and (10) trap flowers (a bowl or more complicated tubular structure with steep and smooth surface upon which insect visitors are “trapped” for some time)

**Pollination type**

To assess potential coevolutionary processes between nectar sugar composition and groups of pollinators (with different energetic demands), we used Müller (1881)‘s flower class as available from BiolFlor (Klotz *et al.* 2002) to define pollination types based on the most predominant pollinator groups visiting the flowering plants: (1) Group ”G” (generalized flowers which can attract a wide range of pollinators, including bees, wasps, flies and syrphids), combined from original groups “A” (flowers with open nectar), “AB” (flowers with partly hidden nectar), “B” (flowers with totally hidden nectar), “B`” (flower heads with totally hidden nectar), “ABDe”, “AD”, “ADe”, “B`F”, “BD”, “BH” and “BHb”; (2) Group ”H” (hymenopteran flowers), combined from original groups “H” and ”HFt”; (3) Group “B” (bee flowers) from original Group “Hb”; (4) Group “BB” (bumblebee flowers) combined from groups “Hh”, “HhDs”, “HhF” and “HhFt”; (5) Group “BF” (butterfly flowers) combined from groups “F”, “Ft”, “FD” and “FHh”; (6) Group “M” (moth flowers) combined from groups “Fn” and “FnH”; (7) Group ”F” (fly flowers) combined from groups “D”, “De”, “Dke”, “Dkl”, “Dt” and “Ds”; (8) Group ”O” (others such as pollen flower and wasp flower), combined from groups “Hw”, “Po”, “BHw” and “Hi”

**Supplementary tables**

**Table S1** A gradient program used at a flow rate of 0.2 mL min^-1^ for measurement of nectar sugar composition.

| Time | Eluent A (12 mM NaOH) | Eluent B (150 mM NaOH) |
| --- | --- | --- |
| 0 | 100 | 0 |
| 12 | 100 | 0 |
| 12,1 | 0 | 100 |
| 25 | 0 | 100 |
| 25,1 | 100 | 0 |
| 35 | 100 | 0 |

**Table S2** Proportion of habitat types at 16 study sites in Saxony-Anhalt, Germany, characterised at a radius of 1 km around the site centre. Habitat mapping was done manually with GIS software (ArcGIS Pro 3.1.41833 ESRI) based on satellite imagery (World Imagery, ESRI) and complemented by field visitation. The classification of habitats is based on an aggregation of the EUNIS habitat classification and comprises 12 habitat types (the main 7 classes are shown): arable = mixed and unmixed crops, bare tilled and fallow land; int. grass = intensively managed/agriculturally-improved grassland; ruderal = road/rail networks, abandoned gardens/fields/constructions/urban areas; urban = buildings; garden = ornamental/domestic gardens, cemeteries; grassland = semi natural grasslands, wood. deciduous = deciduous woodlands/forests.

|  | habitat type (%) | | | | | | |
| --- | --- | --- | --- | --- | --- | --- | --- |
| site | arable | int. grass | ruderal | urban | garden | grassland | wood.  deciduous |
| 1 | 96.88 | 0.00 | 2.28 | 0.04 | 0.00 | 0.15 | 0.65 |
| 2 | 92.44 | 0.00 | 2.63 | 0.32 | 0.32 | 1.32 | 2.96 |
| 3 | 83.35 | 0.00 | 1.96 | 0.05 | 0.00 | 5.19 | 8.83 |
| 4 | 68.32 | 0.12 | 4.52 | 0.74 | 0.72 | 6.20 | 14.90 |
| 5 | 61.95 | 0.00 | 2.36 | 0.26 | 0.51 | 31.06 | 3.68 |
| 6 | 56.98 | 0.19 | 4.50 | 4.40 | 6.28 | 10.43 | 15.30 |
| 7 | 56.03 | 0.00 | 3.16 | 0.81 | 1.51 | 5.27 | 1.00 |
| 8 | 47.56 | 0.00 | 5.68 | 0.74 | 1.31 | 8.55 | 1.67 |
| 9 | 42.65 | 0.00 | 3.64 | 0.81 | 2.72 | 15.14 | 6.24 |
| 10 | 39.53 | 0.00 | 3.81 | 0.52 | 0.22 | 15.52 | 5.71 |
| 11 | 29.44 | 0.00 | 4.46 | 0.05 | 0.00 | 18.84 | 47.20 |
| 12 | 22.41 | 0.00 | 10.48 | 7.01 | 5.13 | 15.98 | 35.53 |
| 13 | 6.07 | 0.70 | 19.28 | 29.32 | 22.05 | 2.64 | 19.39 |
| 14 | 0.64 | 0.15 | 12.49 | 36.18 | 24.32 | 0.24 | 20.83 |
| 15 | 0.00 | 0.00 | 31.04 | 34.36 | 19.59 | 0.00 | 14.99 |
| 16 | 0.00 | 0.49 | 16.17 | 32.43 | 31.06 | 0.19 | 19.66 |

**Table S3** Empirical data of nectar sugar composition, floral tube length and visitation meta-network. Nectar sugar composition of 94 plant species, flower corolla tube length of 388 plant species and aggregated interactions of 48 visitation networks were included.

**Supplementary figures**


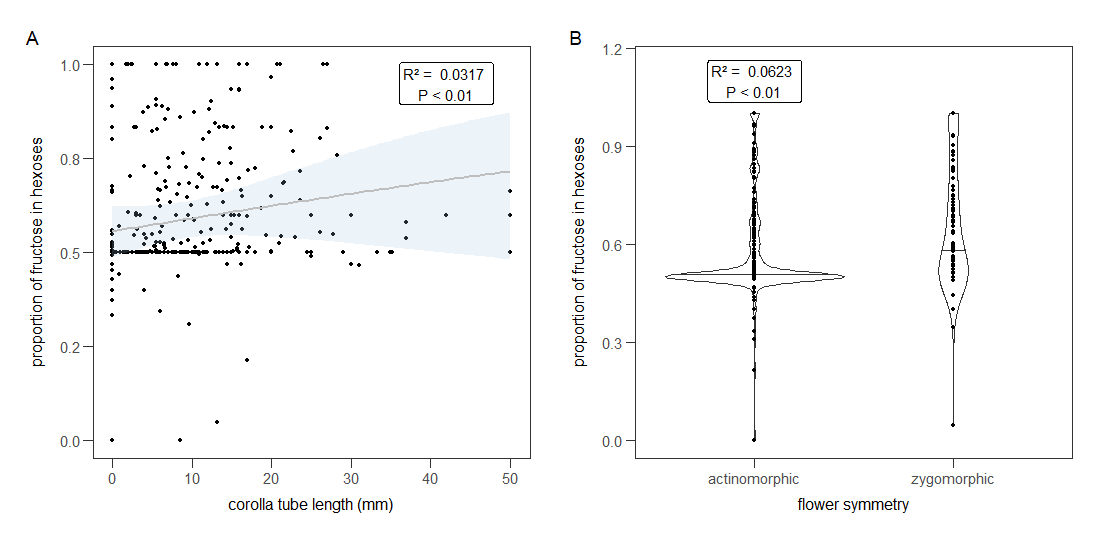


**Fig. S3** Relationship between the proportion of fructose within hexoses and plant corolla tube length (A), and flower symmetry (B). Solid line indicates the predicted relationship and shaded area represents the 95% confidence intervals.

**Additional results based on the dataset excluding our own, empirically derived nectar data**


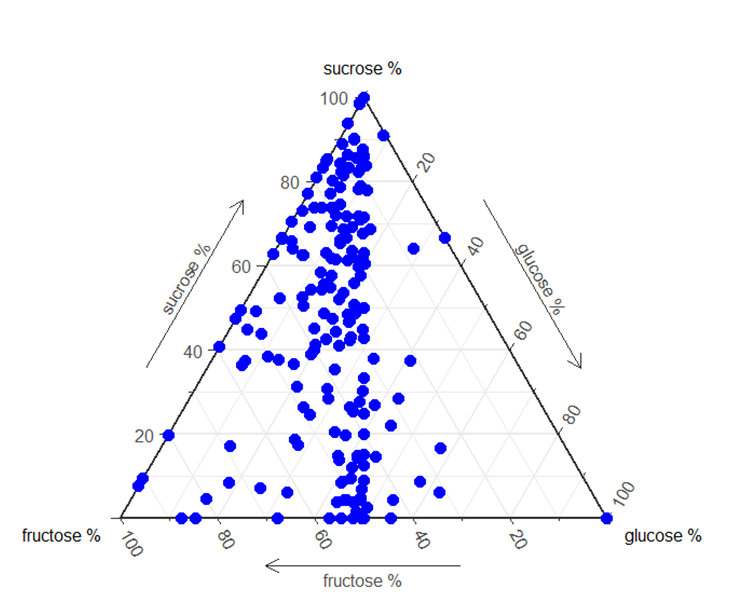


**Fig. S4** Ternary diagram of nectar sugar composition of 353 Central European plant species.


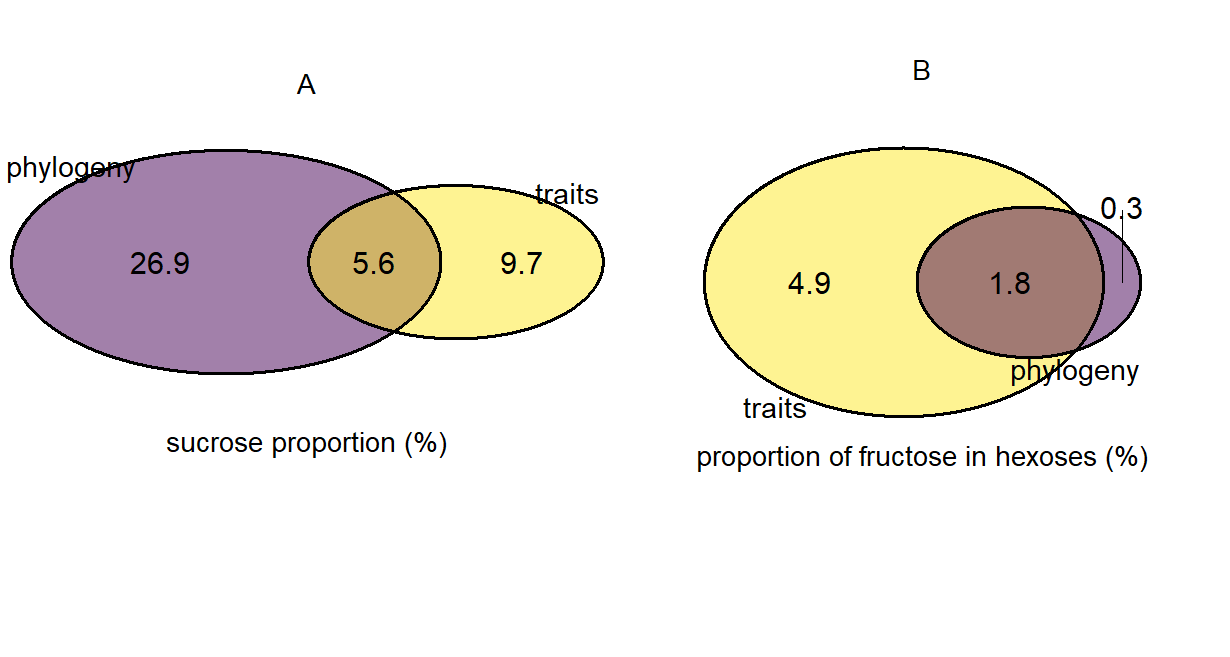


**Fig. S5** Venn diagrams representing partition of the variance in the proportion of sucrose in nectar (A) and the variance of the proportion of fructose within hexoses (B) explained by trait (yellow circle), phylogeny (purple circle) and phylogenetically structured traits (overlapped circles). Note: the sizes of the ovals are not strictly proportional.


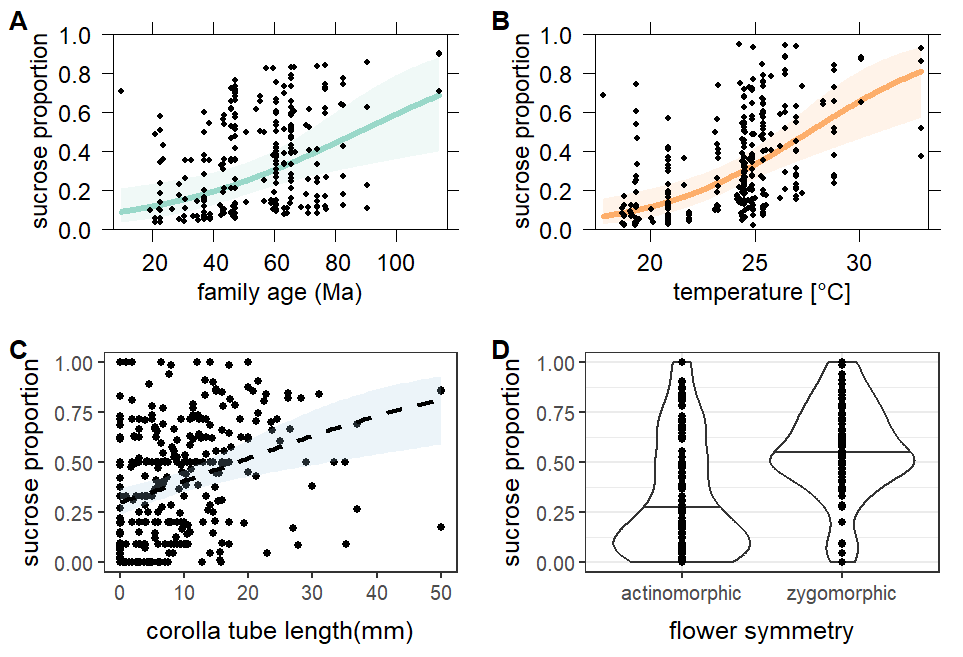


**Fig. S6** Relationship between the proportion of sucrose in nectar and plant family age modelled with plant phylogenetic order as a random effect (A), and global mean annual surface temperature during the time of the origin of plant families, i.e. from Cretaceous until nowadays (B), plant corolla tube length (C), and flower symmetry (D). Solid line indicates the predicted relationship (at the response scale) and shaded areas represents the 95% confidence intervals.

The proportion of sucrose in nectar was phylogenetically conserved and showed a positive relationship with the phylogenetic age of the plant family (slope = 0.021, p < 0.001, AIC = 415.73), but less so with the age of the plant order (p = 0.013, AIC = 425.45). Including plant family nested in plant order as random effects increased model performance and the strength of the positive relationship (Fig. S6 A, slope = 0.029, p < 0.001, AIC = 393.9).

**References**

Klotz, S., Kühn, I., Durka, W. & Briemle, G. (2002) *BIOLFLOR: Eine Datenbank mit biologisch-ökologischen Merkmalen zur Flora von Deutschland*. Bundesamt für Naturschutz Bonn.

Kugler, H. (1970) *Blütenökologie*. G. Fischer.

Müller, H. (1881) *Alpenblumen, ihre Befruchtung durch Insekten und ihre Anpassungen an dieselben*. W. Engelmann.

Percival, M.S. (1961) Types of nectar in angiosperms. *New Phytologist***,** 235-281.
